# Host-directed therapy targeting the *Mycobacterium tuberculosis* granuloma: a review

**DOI:** 10.1007/s00281-015-0537-x

**Published:** 2015-10-28

**Authors:** Dilara Kiran, Brendan K. Podell, Mark Chambers, Randall J. Basaraba

**Affiliations:** Department of Microbiology, Immunology and Pathology, Metabolism of Infectious Diseases Laboratory and Mycobacteria Research Laboratories, College of Veterinary Medicine and Biomedical Sciences, Colorado State University, 200 West Lake Street, 1619 Campus Delivery, Fort Collins, CO 80523-1619 USA; Department of Bacteriology, Animal and Plant Health Agency (APHA), Woodham Lane, New Haw, Addlestone, Surrey KT15 3NB UK; School of Veterinary Medicine Faculty of Health and Medical Sciences, University of Surrey, Vet School Main Building, Daphne Jackson Road, Guildford, GU2 7AL UK

**Keywords:** Tuberculosis, Host-targeted therapy, Granuloma, Metabolism, Glycation, Drug therapy, Adjunctive therapy

## Abstract

Infection by the intracellular bacterial pathogen *Mycobacterium tuberculosis* (Mtb) is a major cause of morbidity and mortality worldwide. Slow progress has been made in lessening the impact of tuberculosis (TB) on human health, especially in parts of the world where Mtb is endemic. Due to the complexity of TB disease, there is still an urgent need to improve diagnosis, prevention, and treatment strategies to control global spread of disease. Active research targeting avenues to prevent infection or transmission through vaccination, to diagnose asymptomatic carriers of Mtb, and to improve antimicrobial drug treatment responses is ongoing. However, this research is hampered by a relatively poor understanding of the pathogenesis of early infection and the factors that contribute to host susceptibility, protection, and the development of active disease. There is increasing interest in the development of adjunctive therapy that will aid the host in responding to Mtb infection appropriately thereby improving the effectiveness of current and future drug treatments. In this review, we summarize what is known about the host response to Mtb infection in humans and animal models and highlight potential therapeutic targets involved in TB granuloma formation and resolution. Strategies designed to shift the balance of TB granuloma formation toward protective rather than destructive processes are discussed based on our current knowledge. These therapeutic strategies are based on the assumption that granuloma formation, although thought to prevent the spread of the tubercle bacillus within and between individuals contributes to manifestations of active TB disease in human patients when left unchecked. This effect of granuloma formation favors the spread of infection and impairs antimicrobial drug treatment. By gaining a better understanding of the mechanisms by which Mtb infection contributes to irreversible tissue damage, down regulates protective immune responses, and delays tissue healing, new treatment strategies can be rationally designed. Granuloma-targeted therapy is advantageous because it allows for the repurpose of existing drugs used to treat other communicable and non-communicable diseases as adjunctive therapies combined with existing and future anti-TB drugs. Thus, the development of adjunctive, granuloma-targeted therapy, like other host-directed therapies, may benefit from the availability of approved drugs to aid in treatment and prevention of TB. In this review, we have attempted to summarize the results of published studies in the context of new innovative approaches to host-directed therapy that need to be more thoroughly explored in pre-clinical animal studies and in human clinical trials.

## Form and function: an introduction to the TB granuloma and granuloma pathogenesis

**Fig. 1:**
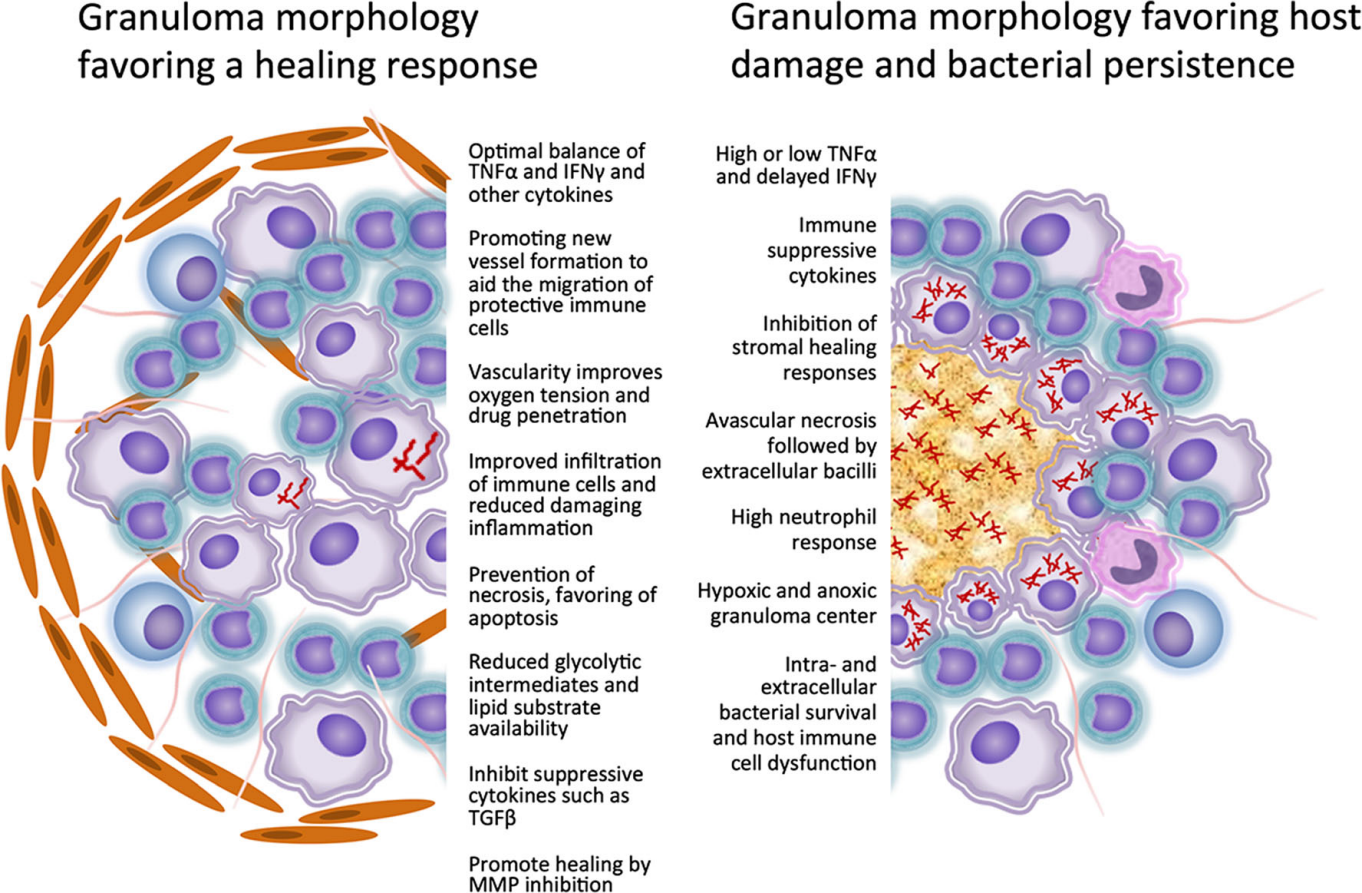
Granulomatous inflammation in response to *M. tuberculosis* infection can be protective or destructive. The typical host response to Mtb infection is infiltration of mixed inflammatory cells at the site of primary infection of macrophages in the lung. The inflammatory response is thought to be necessary to effectively kill bacilli or to prevent the spread of infection within or between hosts. The network of cellular and humoral mediators is complex and a balanced response is necessary to favor a protective response rather than a detrimental response that can result in extensive tissue damage, bacterial persistence, and poor antimicrobial treatment responses. Based on our current knowledge, a number of therapeutic targets can be identified to not only promote a more protective response but to also limit tissue damage. These processes can be promoted or inhibited with existing drugs that can be used alone or as adjunctive treatment in combination with antimicrobial drugs

*Mycobacterium tuberculosis* (Mtb), the primary causative agent of human tuberculosis (TB), remains a prominent global health concern, despite a decline in total incident cases and mortality within the last decade [1]. Currently, the lack of an effective vaccine, reliable diagnostic tests or biomarkers for latent disease, a limited number of effective antimicrobial drugs, and the ongoing emergence of multi-drug resistant Mtb strains continue to challenge current global TB control efforts. As a result, there is increasing interest in novel therapeutic strategies, which improve the host response to Mtb infection and can be used either alone or in combination with conventional and future antimicrobial drug treatments. Gaining a better understanding of the factors that contribute to protective host responses to Mtb infection will aid in the discovery of novel therapeutic targets as well as identify approved drugs that can be repurposed to improve TB treatment and control (Fig. 1).

**Fig. 2:**
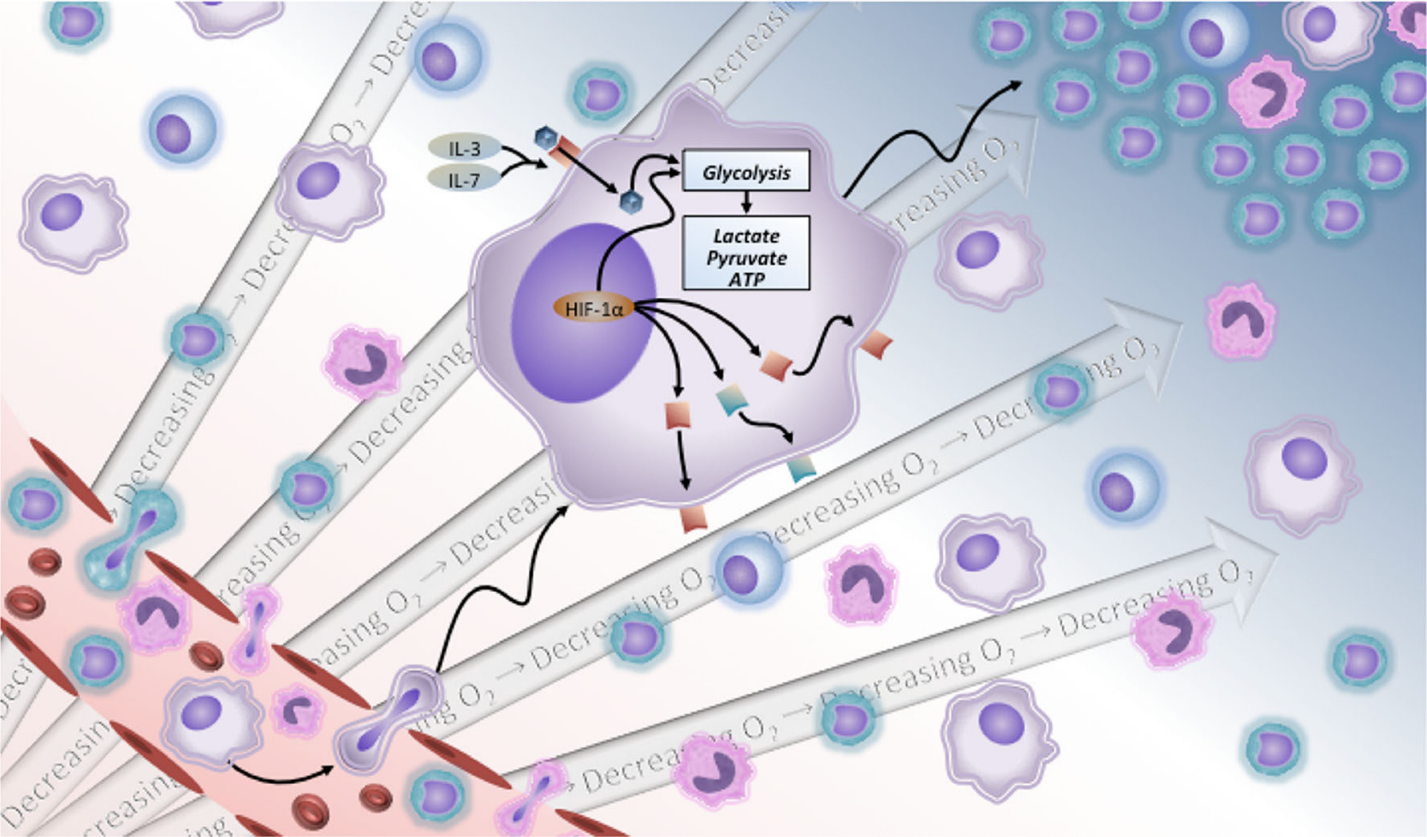
Immune cells responding to *M. tuberculosis* infection undergo a metabolic shift. Like other inflammatory diseases, the mixed population of myeloid cells that respond to Mtb infection undergo a metabolic shift from oxidative phosphorylation to glycolysis in order survive and function effectively within the tissue spaces. As cells leave the high oxygen environment within the blood vasculature, they enter a region of low oxygen tension. This metabolic shift is necessary to function as immune effector cells in the early stages of infection but can be detrimental in a failed immune response associated with active and progressive TB. Intra and extracellular bacilli are able to exploit the host metabolic shift to not only survive an aggressive adaptive immune response but also antimicrobial drug treatment. The changes in both host and pathogen metabolism can be treated using host-directed therapy targeting granuloma formation and resolution

The host response to Mtb infection can be broadly characterized as chronic granulomatous inflammation, which is thought to contain bacilli at the site of infection and prevent dissemination within and between susceptible individuals. The clinical manifestations of active Mtb infection are complex and include an aggressive cellular and humoral immune response aimed at eradicating difficult to kill bacilli as well as clearing persistent bacterial antigens [2, 3]. However, protective granuloma formation can become dysregulated, resulting in an unfavorable inflammatory response and subsequent extensive tissue damage. Irreversible host tissue damage, as evidenced by lesion necrosis and cavitation, contributes to the persistence of drug-tolerant organisms as well as contributes to the spread of infectious bacilli among susceptible individuals [4]. Unregulated inflammation not only results in a failure to clear infection but also impairs host immunity and cellular and systemic metabolic homeostasis. This imbalance between protective and destructive host responses accounts for the variability in the clinical presentation of patients with active TB disease and the high incidence of latent infections. As a result, host-directed therapies, which target granuloma formation and function, should seek to establish a balance between the protective and destructive nature of this typical response to Mtb infection.

The factors involved in the control of TB granuloma formation are poorly understood, but involve adaptations by both the host and pathogen to a changing microenvironment. Alveolar macrophages are thought to be among the first and most important innate host defenses at the site of primary infection in the lung and the most important effector cells encountered by Mtb following aerosol exposure. It is generally believed that the ability of bacilli to survive intracellular killing by macrophages is the initial step in the establishment of Mtb infection [5]. Mtb parasitizes host cells first as a strategy to avoid innate immune surveillance and second as a favorable site to replicate even in the face of an aggressive adaptive cellular immune response. As a consequence, much attention has been given to modulating early and late responses to either prevent the establishment of infection or to control infection following bacterial colonization. In addition, resident macrophages are instrumental in promoting or orchestrating the early inflammatory responses and therefore have a role in establishing a protective or destructive response. If the early host defenses fail to eradicate or significantly slow the growth of Mtb, macrophages contribute to the complicated and dynamic cytokine- and chemokine-mediated recruitment of additional inflammatory cells, which form the early infectious lesion [6, 7].

With the establishment of Mtb infection, expansion of lesions, and progression of disease, granulomas enlarge and undergo alterations in their morphological features. These processes increase the potential for bacilli to spread from the primary site of infection, the lung, to other tissues and organs. During the progression of the inflammatory response, particularly within the relatively high oxygen concentrations in the lung, both Mtb bacilli and host cells undergo a variety of metabolic adaptations in response to immune stimulation. In vitro model systems have clearly demonstrated how limiting oxygen levels alters Mtb metabolism and drug antimicrobial susceptibility [8, 9]. However, very little is known about how the changing microenvironment present during active TB disease in both humans and animals influences host cells and what impact metabolic changes have on immune effector functions. While the role early granuloma formation plays in eradicating bacilli following Mtb infection is unclear, clinical manifestations of active TB disease have been directly linked to a progressive localized and systemic inflammation in response to persistent bacilli or Mtb antigens [10]. Progression of active TB disease, characterized by an increase in granuloma size, number, and distribution, reflects an inability of the host to effectively eliminate bacilli or clear shed antigens and thus is indicative of a failed or ineffective immune response. As a consequence, during the late stages of active disease, Mtb survives despite increased immune cell infiltration, ultimately contributing to granuloma formation and organization. Similar to the early stages of infection, Mtb persistence during the chronic stages of disease is facilitated by the ability of bacilli to acquire and utilize host micronutrients and host-derived intermediates of metabolism needed to survive and replicate [11].

Another hallmark of progressive TB disease, aside from organized granuloma formation, is the development of lesions with central necrosis and cavitation. The appearance of lesions with these morphotypes represents an important transition toward not only irreversible tissue damage in some patients but also the establishment of extracellular populations of Mtb. Extracellular Mtb populations are further isolated from immune effector cells and are functionally tolerant of antimicrobial drug treatment. Most granulomatous lesions share fundamental structural similarities, but differences in the rate of progression and transformation accounts for granuloma heterogeneity within and between patients, equating to variable disease progression and response to treatment. Shown in humans and recently in animal models, heterogeneous patterns of disease evolve as a result of differences in Mtb virulence, host immune status, and the absence of communicable and non-communicable TB risk factors [12–15]. With the development of lesion necrosis and cavitation, some bacilli transition from an intracellular to an extracellular environment concurrently with death and lysis of Mtb infected cells. Other factors, such as the loss of blood supply to expanding granulomas, also contribute to cellular necrosis.

After the release of intracellular bacilli from infected cells, extracellular organisms become attached or enmeshed within a complex matrix composed of host- and pathogen-derived macromolecules. The attachment and colonization of Mtb within these complex extracellular polymeric substances has been equated to biofilm formation, similar to other pathogenic bacteria. However, this concept remains controversial [7, 16, 17]. The adoption of a biofilm-like mode of existence as a consequence of host cell death could explain in part the ability of Mtb bacilli to survive within the harsh granuloma microenvironment as well as its ability to tolerate antimicrobial drug treatment [18]. This view suggests that host cell necrosis as a progression of the host response to Mtb infection is unfavorable rather than protective and gives extracellular Mtb in particular a distinct survival advantage. Although differences likely exist between biofilms at the air-tissue interface and those within caseous necrotic granuloma lesions, information taken from studies of other pathogenic biofilm forming bacteria may lend insight into the development of a drug-resistant Mtb phenotype. The classification of host responses as favorable or unfavorable is a concept that evolved from extensive pathological studies of human TB patients by Canneti and others, which correlated lesion morphology with the ability to isolate viable bacilli by culture [2, 19]. In theory, pharmacological manipulation of the host response to Mtb infection aimed at limiting unfavorable host responses as well as promoting more effective natural immunity could be used as an adjunctive therapy along with antimicrobial drugs. However, studies that focus on host-directed therapy specifically targeting granuloma formation are lacking due to poor understanding of the protective role of early granuloma formation and the factors that determine whether the response to Mtb infection will be protective or destructive. Although the functional role of the granuloma is still under debate, there are a number of therapeutic targets that can be directed at granuloma formation and resolution based on our current knowledge.

## Protective or destructive: the spectrum of granuloma function

Currently, two major frameworks exist around the discussion of the role of the granuloma in TB pathogenesis and treatment. The granuloma is either considered a critical component of the protective cellular immune response, serving a vital role in pathogen containment, or is considered detrimental, contributing to the clinical manifestations of active TB disease and persistence of Mtb. The protective view of granuloma formation stems from the predominance of macrophages and T and B lymphocytes at the site of infection, which are thought to have important effector functions against intracellular Mtb. These cellular infiltrates along with fibrous encapsulation are thought to form a mechanical and functional barrier to prevent bacilli dissemination within and between hosts [20]. However, there is mounting evidence that among Mtb survival strategies, the pathogen has undergone evolutionary adaptations which enable bacilli to persist in the face of this complex host response. These physiological and morphological adaptations not only enable bacilli to subvert host immunity but also allow bacilli to survive for long periods of time within an intracellular and extracellular microenvironment. This prolonged bacillary survival contributes to the development of latent TB disease as well as the expression of antimicrobial drug tolerance [21]. Among the most widely studied adaptations is the transition of bacilli from actively replicating to a state of non-replicative persistence in response to decreasing oxygen concentrations. Classical studies by Wayne and others used in vitro model systems in an attempt to mimic the gradual decrease in oxygen tension, which occurs in vivo as granulomas form, expand in size, and develop necrosis. However, it is likely that a decrease in oxygen concentration is just one of many environmental factors that change as the host response to Mtb evolves in vivo, which remain difficult to measure directly [22–26].

The debate over the protective or destructive result of granuloma formation has thus driven recent investigations into the use of host-directed and specifically granuloma-targeted therapy to either promote natural immunity and healing or to limit the tissue damaging consequences of advanced granuloma formation. Excessive pro-inflammatory responses are unfavorable in the early stages of infection because they result in extensive tissue damage prior to the development of Mtb-specific adaptive immunity. This leads to unregulated inflammation and further tissue damage, creating a microenvironment which promotes the persistence of non-replicating bacilli. In contrast, killing of intracellular bacilli by macrophages as well as maintaining a balanced inflammatory response early during infection contributes to the establishment of more effective adaptive immune protection. Studies have suggested that tight regulation of the cytokines interferon gamma (IFNγ) and tumor necrosis factor alpha (TNFα) is important in coordinating protective granuloma formation [27–29]. Studies also implicate these cytokines in the maintenance of granuloma structure, and when inhibited, lead to a loss of granuloma integrity and subsequent reactivation and spread of Mtb. However, it is unclear whether the loss of granuloma structure is a consequence of specific cytokine deficiencies or whether disorganized granulomas are due to a new wave of acute inflammation in response to reactivated bacterial growth [30].

The cytokine TNFα in particular has a dose-dependent effect on lesion morphology [29]. The absence of TNFα leads to an unregulated inflammatory response and uncontrolled bacterial growth, whereas excess TNFα has been to shown to compromise lung function [31]. Recent data suggests that TNFα may also have a direct effect on Mtb or infected macrophages given that anti-TNFα antibody treatment reactivates bacterial growth in an in vitro granuloma model. Under these experimental conditions, in vitro granulomas, which are essentially peripheral blood mononuclear cells clustered around infected cells, lack the structural complexity ascribed to the ability of in vivo granulomas to contain infection [32]. These data argue against the functional significance of granuloma integrity in the control of Mtb growth and dissemination. However, a balanced concentration of TNFα results in smaller, more organized lesions [33]. This dose- or time-dependent cytokine response has been referred to as the “goldilocks effect”: severe, pro-inflammatory responses in the early stages of infection may benefit the bacteria by promoting more extensive tissue damage. In contrast, delayed or incomplete granuloma formation may leave the host un-protected, thus favoring unrestricted bacterial growth and dissemination in the late stages of disease [34–36]. This balance between pro- and anti-inflammatory mediators has been linked to a hypersusceptible phenotype in human populations in Vietnam, where polymorphisms in the leukotriene A4 hydrolase (LTA4H) lead to either high or low enzymatic activity and worse disease. In the zebrafish TB model, the lipid mediator by-product of LTA4H, leukotriene B4, is critical in impairing anti-inflammatory lipoxins and promoting a balanced production of TNFα, which limits high intracellular bacterial loads and cellular necrosis [37, 38]. Based on this conceptual framework, limiting TNFα production therapeutically in the early stages of Mtb infection of patients prone to a pro-inflammatory response could be beneficial. Unfortunately, peripheral blood levels of this and other cytokines may not reflect the dynamic change in cytokine concentrations at the site of infection, and therefore are not reliable biomarkers on which to base the timing of therapeutic intervention [39–41]. Recent studies have demonstrated that besides limiting the effects of TNFα through the use of anti-cytokine antibodies, other drugs such as metformin, a compound used for the treatment of type 2 diabetes, have off target effects that include reducing TNFα levels in mice and in an in vitro human monocyte model. Therefore, metformin has recently been considered for re-purpose in TB treatment [42–44]. Reducing cytokine-mediated inflammation may assist the natural host immune responses in clearing Mtb infection while at the same time maintaining a more balanced inflammatory response, especially during early granuloma formation.

## Then and now: a historical perspective of granuloma targeted therapy

Historically, treatments specifically targeting the TB granulomas consisted of surgical-based techniques, practiced most commonly in the pre-antibiotic era. TB patients that were considered candidates for surgical intervention generally shared in common lesions with extensive tissue damage often with lesion necrosis or cavitation. Collapse therapy was among the first interventional adjunctive granuloma-targeted therapies with the potential to improve patient survival and overall TB treatment outcomes. Collapse therapy encompassed many procedures, including pneumothorax (decreasing intra-thoracic negative pressure via introduction of air into the pleural space), pneumoperitoneum (introduction of air or gas into the abdominal cavity), phrenic crush (sectioning of the phrenic nerve to induce partial or complete diaphragm paralysis), thoracoplasty (removal of the ribs from the chest wall), and extrapleural lucite pack (surgical addition of inert lucite substance into lung cavities to induce collapse) [45]. These treatments resulted in partial collapse of the lung parenchyma as a strategy to reduce dead air spaces within TB cavities. The overall goal of collapse therapy was to promote healing of extensively damaged lung parenchyma [46]. The principle is based on the fact that by collapsing the lung and associated cavitary lesions, more normal tissue margins become closely apposed, which enables the reestablishment of blood supply and therefore the promotion of wound healing and resolution. However, complications such as pleural adhesions and thickening, bronchial fistulae, and secondary bacterial infections limited the therapeutic value of this practice [47].

Surgical lung re-sectioning of portions or entire lung lobes referred to as a lobectomy is also a common adjunctive treatment, particularly in patients that fail to respond to conventional antimicrobial therapy. The overall goal of removing entire lung lobes is to physically reduce the bacterial and lesion burden and therefore regions of the lung that were most refractory to antimicrobial treatment. Similar to collapse therapy, surgical debulking of damaged tissue aids in re-establishing vascular perfusion and healing of more normal tissue to improve drug treatment responses and patient survival. The development of technological advances including video-assisted thoracoscopic approaches enable more efficient and selective removal of lesions while at the same time reducing the incidence of postoperative complications [48]. With the increasing occurrence of multi-drug-resistant (MDR) and extensively drug-resistant (XDR) Mtb infections, which fail to respond to antimicrobials drug combinations, lung resections are still considered safe, viable options to decrease the overall lesion burden and the majority of drug-resistant bacteria [49]. Patients infected with MDR and XDR strains of Mtb can also benefit from surgical-based adjunctive treatment in the early stages of infection to avoid disease progression in patients, which would otherwise be untreatable [50].

Corticosteroids were explored as a potential host-directed therapeutic based on their anti-inflammatory properties. Recent meta-analysis indicates that 17 % of TB-associated mortality can be reduced with the use of corticosteroid therapy. However, when sensitivity analysis was conducted by the same authors on cases of pulmonary TB alone, there was no significant reduction in mortality when corticosteroids were used [51]. Prospective controlled trials have shown that corticosteroid use is beneficial in the treatment of meningitis, pericardial, and pleural TB disease. However, clinical and radiographic improvements of pulmonary TB observed in early stages of corticosteroid treatment were lost at 6 months of use [52]. Mice infected with the H37Rv strain of Mtb strain and treated with corticotrophin, cortisone, or hydrocortisone displayed a significant increase in microbial populations in the lungs and spleen, had shorter survival times compared to untreated mice, and possessed altered lesion morphology [53, 54]. Recently, it has been shown that corticosteroids, such as dexamethasone, significantly reduce cytokine responses to TB antigens and interfere with diagnostic assay results [55]. Utilization of oral or inhaled corticosteroids by individuals with other forms of respiratory illness, such as asthma or COPD, has also been shown to increase the risk of TB infection in a dose-dependent manner [56, 57]. Therefore, while anti-inflammatory properties of corticosteroids may be beneficial in controlling TB in certain stages of disease and in certain locations, the immunosuppressive functions of corticosteroids appear to hinder the clearance of Mtb.

The discovery of the anti-TB drug isoniazid (INH) led to some of the first studies suggesting that poor antimicrobial treatment responses were in part due to poor drug penetration [58]. These studies were designed to determine what effect lesion morphology had on antimicrobial drug penetration in human TB patients and guinea pigs with experimental Mtb infection [58–60]. Barclay et al. using ^14^C labeled INH, and others more recently using advanced mass spectrometry-based techniques, demonstrate that antimicrobial drug penetration is severely impaired in TB lesions with necrosis or cavitation [61, 62]. As a result of these historical perspectives, new technology is being used to gain a better understanding of pharmacokinetics, bioavailability, and penetration of antimicrobial drugs alone and in combination [63]. The heterogeneity of TB granulomas accounts for the measured differences in antimicrobial drug penetration and accumulation, a feature that contributes significantly to poor treatment responses in humans and animal models [63]. In the C3HeB/FeJ mouse model and in rabbits, antimicrobial drugs had reduced activity in large, caseous lesions [12, 13]. This could be in result to multiple factors such as the size of the lesion, specific drug combinations utilized, variation in fibrosis, and level of caseation. In this way, the necrotic granuloma has been shown to be a mechanical and functional barrier to anti-microbial treatment, which in part explains the benefit of surgical intervention as an adjunctive therapy. Recently, computational models have been used to integrate pharmacokinetics, pharmacodynamics, and specific morphologic features of granulomatous lesions to illustrate the potential benefits of host-directed therapies specifically targeting the granuloma [64]. The use of this approach will help gain additional knowledge about the bioavailability of existing drugs and drug combinations and will aid in the development of more effective drugs in the future. Importantly, investigational studies taking this approach can assess the potential benefits of strategies to increase intra-granuloma drug penetration of new experimental drugs or drugs designed to increase blood flow by promoting new vessel formation [65].

## The therapeutic potential of granuloma angiogenesis

The inability of antimicrobial drugs to reach therapeutic levels within granulomatous lesions not only accounts for poor treatment responses but also has the potential to promote the development of drug-tolerant Mtb phenotypes and contribute to the emergence of drug-resistant strains of Mtb. The failure of antimicrobial drugs to reach therapeutic concentrations may have a similar effect to improper dosing or patient non-compliance. Therefore, an additional host-targeted therapy aimed at promoting granuloma vasculature perfusion also has the potential to improve drug treatment responses. Since oral or parenterally administered drugs must first reach therapeutic blood levels, there is a direct correlation between peripheral blood drug levels and what can be achieved within the TB lesion. As a consequence, lesion vascular integrity and blood perfusion have the potential to significantly impact therapeutic effectiveness of antimicrobial drugs. Granuloma targeted therapy can be designed to either promote or inhibit new vessel formation (angiogenesis) depending on the stage of disease and the overall treatment objective. The potential of granuloma therapy targeting blood vasculature is best illustrated from the advances made in the treatment of cancer. Although no live pathogen is associated with cancer development in this case, the gap in knowledge regarding immunological and metabolic changes that occur within the granuloma microenvironment necessitates comparisons to be drawn from this area of study.

Inhibition of tumor microvasculature serves as the basis of numerous cancer-based therapies designed to inhibit tumor-associated new vessel formation, which contributes to the growth and spread of neoplastic cells. Since tumor metastasis requires an intact blood supply, and neoplastic cells require the expression of proangiogenic factors to grow and spread, one approach to cancer therapy is to restrict growth and metastatic potential by starving tumors of oxygen and nutrients through inhibiting angiogenesis [66]. Despite the similarities between cancer and TB granuloma formation, a therapeutic strategy designed to promote rather than inhibit granuloma angiogenesis would seem to have the greatest therapeutic benefit. Restoring granuloma blood supply would not only have the potential to improve antimicrobial drug treatment responses but would also restore more normal tissue oxygen concentrations, promote healing, and facilitate more effective infiltration of effector immune cells to sites harboring persistent, non-replicating bacilli. However, in the absence of antimicrobial drug treatment, reestablishing lesion perfusion could be detrimental by facilitating bacilli dissemination. Therefore, granuloma-targeted therapy aimed at restoring vascular perfusion would likely only be used as an adjunctive treatment along with bactericidal or bacteriostatic drugs [65].

A notable similarity between the tumor and granuloma microenvironment is localized hypoxia and the development of central lesion necrosis. In both cases, the development of central necrosis is in part due to the inability of tumor and granuloma vasculature to keep up with expansible growth of the lesions relative to the more normal surrounding tissue. In addition, the disruption of lesion vasculature is also due in part to the procoagulant state associated with chronic inflammation [67, 68]. As mentioned above, in the case of TB granuloma formation, components of the Mtb cell wall and secreted proteins have the potential to also contribute to the development of central necrosis [69–71]. Differences in granuloma blood supply have been shown to be associated with differential development of granuloma hypoxia and necrosis in *Mycobacterium avium*-infected mice [27]. Wild-type mice infected with *M. avium* showed alteration of lung vasculature in conjunction with changing tissue architecture, resulting in marked reductions in vessel density within the granuloma center. Additionally, expression of angiogenic factor mRNA is down regulated during mycobacterial infection. Vascular endothelial growth factor (VEGF), a potent inducer of angiogenesis which also increases endothelial cell vascular permeability, has been shown to be increased in the serum of individuals with active pulmonary TB when compared to both inactive TB infection and healthy individuals [72, 73]. Therefore, modulating the presence of these factors may help to prevent the loss of vascularity and thus the formation of caseous necrosis [27]. Although Mtb does not cause the same reduction of vascularization, hypoxia, or granuloma necrosis in the majority of mouse model strains, the guinea pig model of infection has shown similar pathologic features to that of the human and also remains a driving force for the relevance of necrotic lesion-bearing mouse models such as the C3HeB/FeJ strain. These models illustrate the importance of a balance in the expression of angiogenic factors, which is also relevant to host-directed Mtb therapeutics intended for humans [74]. Based on these data, any model, which develops granuloma necrosis or cavitation in response Mtb infection, may benefit from this therapeutic strategy. However, more extensive investigation into this area of research is needed.

The association between granuloma formation and angiogenesis was also shown in the *Mycobacterium marinum* zebrafish embryo infection model. It was demonstrated that macrophages specifically induced the growth of new vessels in the inflammatory environment. However, inhibiting VEGF proved to be beneficial by reducing vascular leakage, and modulating angiogenic factor signal pathways altered oxygen availability for mycobacteria [75]. Similar results were seen when anti-VEGF compounds were utilized in TB patients. Anti-VEGF compounds were capable of normalizing vascular integrity in TB patients, reducing granuloma hypoxia, and promoting small molecule delivery [76]. Reduced angiogenic activity as a result of neutralizing antibodies targeting VEGF was also observed in human mononuclear cells incubated with sera from TB patients [77]. Pazopanib, a VEGF receptor tyrosine kinase inhibitor, currently in clinical trials and shown to be an effective antitumor therapeutic, may prove to be a viable option for a TB therapy targeting granuloma angiogenesis [78]. Pazopanib has been tested in the *M. marinum* infection models and has been shown to reduce bacterial burden, reduce vascular leakiness, decrease Mtb dissemination, and also increase the effectiveness of first line anti-tubercular drug rifampicin [75]. The presence of IL-12p40 and TNFα has also been correlated with the level of sera angiogenic activity, suggesting they may play a role as mediators of angiogenesis [77]. While the zebra fish embryo infection model sheds insight into multiple effects of angiogenic modulators on granuloma development, the risks of vascular disruption secondary to use of pro-angiogenic modulators could be overlooked. Vascular erosion as a consequence of expanding inflammation in a zebrafish embryo will not have the same pathological or clinical impact that pulmonary arterial or venous erosion would have on mammalian animal models or humans. However, the risk of hemoptysis secondary to TB cases is low in the developed world. Only 1.4 % of hemoptysis cases seen at a tertiary referral clinic were due to active TB infection [79]. While the risk of hemoptysis-related complications secondary to TB infection increases in countries where TB is endemic, certain studies show that it may be an indicator of improved survival and has a lower hazard ratio than other TB-related complications [80, 81]. Despite these findings, further research on the use of angiogenic modulators should be conducted in other animal models to determine if there are any in vivo contraindications when used as host-directed therapies.

While therapies inhibiting angiogenesis have shown promise in reducing the rate of granuloma formation and severity, the opposite approach could also be beneficial. Rather than limit angiogenesis in an area of the granuloma that already has restricted access to host blood supply, there is the potential that increasing angiogenesis and promoting the blood supply will allow for more efficient drug penetration and increase the access of host immune cells to the granuloma [65]. One study demonstrated that the expression of VEGF in TB patient sera was correlated with the lack of cavity formation, demonstrating the plausibility of a pro-angiogenic host-targeted therapeutic [82]. An in vitro model of angiogenesis demonstrated that endothelial cells produce increased levels of VEGF, transforming growth factor (TGF), hypoxia inducible factor (HIF), epidermal growth factor (EGF), platelet-derived growth factor (PDGF), fibroblast growth factor (FGF), and bone morphogenetic protein (BMP) growth factors when undergoing angiogenesis [83]. Treatments that promote the transcription of these genes may also promote granuloma angiogenesis, which could be beneficial for the reasons described above. In addition, glucagon-like peptide-1 (GLP-1), a hormone involved in glucose homeostasis, has also been shown to promote angiogenesis in a dose-dependent manner in an in vitro model of human endothelial cells [84]. Based on these data, GLP agonists previously developed for anti-diabetic purposes, such as exenatide, could potentially be utilized as a host-directed therapy targeting the TB granuloma [85]. Endothelial cells are dependent upon glucose and glutamine for proliferation and produce lactate when undergoing glycolysis in hypoxic environments. Lactate inhibits prolyl hydroxylase enzymes and promotes the transcription of pro-angiogenic factors, serving as a driver of angiogenic processes [86]. Since the granuloma represents a hypoxic microenvironment, favoring lactate production via prolyl hydroxylase inhibitors may be an effective mechanism to also promote angiogenesis. Overall, angiogenesis is a promising target for granuloma-directed therapies, and more research is needed to determine if promotion or inhibition of angiogenesis is more efficacious in the context of human Mtb infection.

## The granuloma as an interface for metabolism and immunity

Similar to other inflammatory lesions, TB granuloma formation is characterized by an altered metabolic state both at the cellular and systemic level. The metabolic profile of cells that infiltrate the site of Mtb infection share similarities with the changing metabolic profile of the Mtb organism itself. The nature of the granuloma as an inflammatory lesion makes the metabolism of surrounding immune cells extremely important in the context of clearance of Mtb infection. The complex relationship between host-pathogen metabolism and Mtb infection has been recently reviewed [87]. Altered metabolic features are a result of the environmental stresses found within the granuloma microenvironment, such as limited nutrient availability, hypoxia, and low pH. Mtb infection has been shown to alter the host metabolome, increasing the concentration of metabolites such as d-gluconic acid, d-lactone, glutaric acid, butanal, and ethane within TB-positive sputum samples as a result of increased glucose oxidation, oxidative stress, and lipid peroxidation [88]. Similar metabolic adaptations have been demonstrated in guinea pigs infected with Mtb [89, 90].

In response to cytokine- or antigen-mediated stimulation, immune cells undergo a metabolic shift from oxidative phosphorylation to glycolysis (Fig. 2). Rather than utilize the tricarboxylic acid (TCA) cycle and oxidative phosphorylation, activated immune cells produce energy through the uptake and utilization of glucose for the production of lactate [91]. Much of what is known about the relationship between metabolism and immunity has been learned from the study of other inflammatory diseases and cancer. Similar to the importance of blood supply for tumor and granuloma development, the immune cells that respond to Mtb infection also share important similarities to cancer cells with regards to cellular metabolism [92]. Tumor metabolism is broadly characterized by a metabolic shift from oxidative phosphorylation to aerobic glycolysis even in the presence of adequate oxygen, through a process known as the Warburg effect. Tumor cells are dependent upon aerobic glycolysis for growth and survival [93]. Upon activation, immune cells such as macrophages and neutrophils undergo a respiratory burst during which they increase oxygen consumption, utilize glucose, and produce superoxide radicals. Additionally, molecules that stimulate a respiratory burst, such as PMA and GM-CSF, regulate the production of key glucose transporters [94]. T and B lymphocytes also undergo a metabolic shift in response to cytokine or antigen stimulation which involves the glucose-regulating growth hormone insulin. The insulin receptor is a key cell surface expressed receptor indicative of B and T lymphocyte activation [95]. Similar to other immune cells, lymphocytes responding to an inflammatory stimulus favor lipid oxidation, glutamine catabolism, aerobic and possibly anaerobic glycolysis over glucose oxidation via the TCA cycle [96]. T cell activation is dependent upon glucose uptake via GLUT1 and CD28 stimulation [97]. The emigration of immune cells from the oxygen-rich blood vasculature to the relatively oxygen-deplete tissue space at the site of infection alters cellular metabolism to utilize glucose within an inflammatory microenvironment [35]. Interleukin-3 (IL-3), a key cellular survival factor, has been shown to stimulate the translocation of a key glucose transporter, GLUT1, to the cell surface in a protein kinase-dependent fashion. IL-3 is also directly related to the regulation of apoptosis, as inhibiting glucose uptake via transporters such as GLUT1 enhances apoptosis [98]. As a result, anti-glycolytic agents such as 2-deoxyglucose have been explored as an anti-cancer therapeutic strategy to inhibit glycolysis, the preferred metabolic pathway of tumor cells.

A similar metabolic shift occurs within the TB granuloma and additional environmental changes such as a reduction or lack of oxygen within the necrotic granuloma increase the level of complexity. Although glycolysis is less energy efficient, glycolysis is active under aerobic or anaerobic conditions, enabling immune cells to function even under low oxygen condition [99]. The chronic TB granuloma is characteristically hypoxic as demonstrated in human TB lesions and a variety of animal models, particularly those that develop caseous necrosis [22, 25, 26]. Therefore, glycolysis, glucose transport, and overall glucose homeostasis are key processes that are relevant to host immune cell function during Mtb infection. These metabolic processes are regulated via the phosphatidylinositol 3-kinase (PI3K) pathway and its core kinases AKT, adenosine monophosphate activated protein kinase (AMPK), and TOR [91]. Hypoxia has also been demonstrated to suppress glutamine entry into the TCA cycle, and glutamine-dependent metabolic pathways have been shown to be important cellular survival strategies within hypoxic environments [44]. Immune cells are able to adapt to decreasing oxygen tension via activation of hypoxia-inducible factor 1 (HIF-1), which functions via adenosine receptor signaling pathways [100]. In contrast, excessive glucose uptake can be detrimental to the cell, resulting in activation of pro-apoptotic factors and cell death. As a consequence, the ability to maintain a balance in metabolic homeostasis within immune cells and systemically is critical for a protective immune response to Mtb infection [101].

Tyrosine kinases, key host signaling molecules, which play a role in mycobacterial entry and survival within macrophages, have recently been implicated as targets for host-directed therapeutics. Host tyrosine kinases function as regulators of phagosomal acidification, lysosomal mobility, and autophagy in macrophages and inhibition results in increased acidification of monocyte-derived macrophages [102]. In an infection model of *M. marinum*, the utilization of a tyrosine kinase inhibitor proven successful for antitumor treatments, imatinib, successfully reduced bacterial load, reduced lesion pathology, was effective against rifampicin-resistant *M. marinum* strains, and worked synergistically with rifampicin to reduce bacterial burden. Furthermore, when imatinib was utilized in a murine Mtb infection model, similar decreases in bacterial load were observed [103]. A murine Mtb infection model also demonstrated that orally administered imatinib facilitated a growth reduction of intracellular bacilli [102]. The utilization of imatinib in conjunction with conventional antimicrobials is thus a promising host-directed therapy targeting macrophages within the TB granuloma. Imantinib has also been shown to affect T cell function, inhibiting primary T cell proliferation and reducing expansion of primary cytotoxic T cells (101). In the context of Mtb infection, utilizing imatinib to inhibit certain T cell population expansions may help to down regulate the production of key pro-inflammatory cytokines, which push the balance of the granuloma toward destructive processes.

Metformin is one of the safest and most widely prescribed drugs in the world and, when combined with dietary changes and exercise, helps in maintaining more normal blood glucose levels in patients with type 2 diabetes. Metformin, a biguanide class of anti-diabetic drugs, lowers blood glucose levels via inhibition of gluconeogenesis in the liver by targeting the mitochondrial complex I, thus limiting dysregulation in kinase signal pathways and altered lipid metabolism [104]. Although used for the treatment of diabetes and more recently as an adjunctive treatment for cancer, studies have shown the potential of metformin as an adjunctive TB therapy as well [43, 105]. Metformin has been shown to restrict Mtb growth, reduce tissue pathology and chronic inflammation, enhance the host immune response, and decrease overall TB severity in animal models [43]. This occurs as a result of increased host production of reactive oxygen species, increased acidification of phagosomes containing Mtb, and activation of AMPK-dependent pathways [43]. Further, metformin has demonstrated potential to increase the efficacy of conventional TB treatment options [43, 105]. The early success of metformin in TB animal models points to the targeting of host metabolism, which assists in enhancing protective features of the granuloma. This application also illustrates the potential value of repurposing drugs as adjunctive TB treatment as a viable host-directed therapeutic approach.

Macrophages within the granuloma also have altered lipid metabolism that develops over the course of infection, as reflected by a foamy phenotype resulting from accumulated cytoplasmic lipid droplets and lipid storage organelles [106, 107]. This phenotypic change is accompanied by an up regulation of lipid synthesis and sequestration pathways, and is correlated with granuloma caseation. Triacylglycerol (TAG), cholesterol, and cholesterol esters are the most abundant lipids present in TB granuloma lesions [108]. The accumulation of TAG is also associated with the formation of lipid bodies within foamy macrophages. Macrophages accumulate TAG under hypoxic conditions, and Mtb acquires TAG from these host cells and further utilizes host TAG to synthesize its cell wall lipids [109, 110]. The Mtb TAG synthetic pathway diverts carbon away from the TCA cycle and as a result has been implicated in the development of in vivo drug tolerance. This is supported by evidence that Mtb remains sensitive to antimicrobials if the TAG synthesis pathways are disrupted [111]. Furthermore, Mtb is able to direct fatty acid metabolites toward pathways that prevent their toxic accumulation within the granuloma [11]. Cholesterol esters are typically produced by foamy macrophages; thus, their presence in granulomas is indicative of a degenerative process which may lead to cell death and contribute to the development of caseous necrosis [112]. Labeling of macrophage with proprionate, oleate, and stearate within lipid droplets prior to infection demonstrated that Mtb has access to and incorporates host fatty acids into its cell wall lipids [11]. Mtb is also capable of utilizing host-derived fatty acids, lipids, and cholesterol as alternative carbon sources during infection [87]. The induction of foamy macrophage formation has been linked to decreased host cell glycolytic activity and enhanced ketone body synthesis via G protein-coupled receptor GPR109A feedback [107]. Inhibition of GPR109A resulted in dose-dependent reduction in bacterial survival, a reduction in the number of infected cells, reduced bacillary load, and diminished lipid bodies of alveolar macrophages in vivo [107]. Therefore, G protein coupled receptor inhibitors may be a viable treatment option to target progression of granuloma pathology during Mtb infection. Vitamin D, an essential molecule for IFNγ-mediated antimicrobial activities for macrophages infected with Mtb, was found to reduce the accumulation of lipid droplets in host macrophages and down regulate the proadipogenic peroxisome proliferator-activated receptor γ (PPARγ) [113, 114]. Vitamin D signaling pathways may therefore also be a strategy for host-directed therapies targeting granuloma formation.

As the macrophage becomes glucose deprived, Mtb begins to utilize host lipids as a primary source for metabolic function and alters glycolytic pathways to function under reduced oxygen tension [87, 115]. Mtb will preferentially utilize gluconeogenic carbon substrates, such as fatty acids, during infection [116]. This alternative metabolic pathway under limited oxygen conditions involves up regulation of genes involved in reverse TCA cycle activity and results in the accumulation of succinate [117]. Succinate has been shown to be essential to the adaptation of Mtb to hypoxia, allowing the bacilli to maintain membrane potential and ATP synthesis [118]. Furthermore, isocitrate lyase, an enzyme associated with the glyoxylate shunt and which is capable of producing succinate under alternate metabolic pathways, is required for Mtb growth and virulence in vivo [119]. The host immune response is capable of taking advantage of the bacterium’s need for succinate. Immuno-responsive gene 1 has been discussed as a potential Mtb inhibitor since it produces itaconic acid, an analog to succinate, which is capable of inhibiting isocitrate lyase pathways in activated macrophages [87, 120, 121]. The importance of succinate and its synthesis pathways in the progression of Mtb infection and ability for Mtb to persist inside the granuloma demonstrate potential for host-directed therapies.

## Advanced glycation end products in TB disease

Advanced glycation end products (AGEs) are modified proteins, which have chemically reacted with sugar residues, such as glucose, thus significantly altering protein structure and function [122, 123]. AGEs can function in both receptor-dependent and receptor-independent mechanisms. AGEs have been widely studied in the context of diabetes and hyperglycemia. High glucose concentrations combined with oxidative stress lead to accelerated glycation via generation of intermediate reactive aldehydes, and have been shown to be central in the pathogenesis of diabetic vascular complications. Circulating AGEs are capable of creating more AGEs, which further amplifies their toxic effects [122]. AGEs affect endothelial cell junctions, increasing vascular permeability, and may induce apoptosis in some cases. However, angiopoietin 1, a protective endothelial cell factor with anti-apoptotic properties, has been demonstrated to provide protection to endothelial cells following AGE exposure [124]. The receptor of AGE (RAGE) has been found on the surface of macrophages and endothelial cells and plays an important role in inflammation. Oxidative stress has been linked to AGE production, and increases in reactive oxygen species (ROS) have been positively correlated to increased AGEs and increased glucose. Furthermore, antioxidants such as Resveratrol are capable of ameliorating this effect [125]. In microenvironments with oxidative stress, there are also low levels of the soluble RAGE receptor, which has been shown to have therapeutic potential. Utilizing the soluble form of the RAGE receptor as a competitive binding agent may help reduce circulating AGE concentrations. Anti-AGE compounds, such as pyridoxamine, are being studied in the context of diabetes treatment and could be applied as an adjunctive host-directed therapy targeting the granuloma, based on the aforementioned functions of AGEs in TB disease progression. AGEs contribute to vascular complications via induced dysfunction of endothelial progenitor cells. Rosiglitazone, an agonist of PPARγ, is capable of reversing AGE-mediated inhibition of endothelial cells via the PI3K-AKt-eNOS pathway [126]. GLP-1 receptor agonists, such as exendin-4, have also been shown to repress RAGE expression and subsequently inhibit hyperglycemia-induced apoptosis [127]. Interaction of AGE with RAGE results in an induction of an oxidant stress cascade regulated by MAP kinases [128].

Recent research has revealed that AGEs play a role in Mtb infection and TB disease progression. RAGE is known to be constitutively expressed in the lung and it has been shown that levels of RAGE are elevated in mice infected with Mtb as well as human cells stimulated with Mtb antigens [129, 130]. However, mice which lack the RAGE gene display enhanced inflammation, increased edema, elevated levels of pro-inflammatory cytokines, and increased numbers of leukocytes at the site of infection [130]. These results suggest that RAGE signaling is important in maintaining the balance between a destructive and a protective granulomatous lesion and that up regulating RAGE signaling may help to control chronic infection. The guinea pig model of infection also points to a role of AGEs in TB infection. In a non-diabetic hyperglycemia-induced guinea pig comorbidity model of TB infection, hyperglycemia increased TB disease severity, and Mtb infection induced the formation and accumulation of AGEs in serum as well as within granulomatous lesions [131]. Interestingly, AGE accumulation was shown to increase in Mtb-infected guinea pigs, regardless of whether the animal was dosed with sucrose or water as a carrier control, but sucrose feeding resulted in a greater accumulation of tissue AGEs. In vitro macrophage infection models with BCG also demonstrate AGE accumulation as a result of increased methylglyoxyl, a potent glycating agent, contributing to mycobacteria-induced apoptosis [132]. Generation of AGEs within foci of chronic inflammation may contribute to cell and tissue damage within the TB lesion based on engagement of AGE with the RAGE receptor on endothelial cells surfaces [133].

RAGE has been linked to autophagy and the establishment of neutrophil extracellular traps (NETS) in the context of adenocarcinoma [134]. NETs consist of a complex of neutrophil expelled chromatin, granule proteins, and released ROS and function in a wide variety of pathogenic infections [135]. Mtb has been shown to induce NETs in vitro, and Mtb induced NETs result in a pro-inflammatory activation of macrophages [136, 137]. The enhancement of AGE and RAGE production during Mtb infection may therefore contribute to production of NETs, activation of macrophages, and chronic inflammatory progression of granuloma lesions. As a result, treatment which targets the production of AGEs and RAGE within the granuloma could either serve to enhance macrophage function and accelerate macrophage killing of Mtb or could serve as a mechanism by which the chronic inflammation of advanced TB patients could be reduced. Metformin could be utilized for this purpose, as it is capable of reducing the impact of AGE production via suppression of RAGE in an AMPK-dependent fashion [138].

Currently, there are no FDA approved or available drugs that specifically prevent the formation and accumulation of AGEs in humans or animals. Aminoguanidine has received the most attention to date as a potential anti-glycation therapeutic and is considered the gold standard for anti-AGE activity. Aminoguanidine showed efficacy in animal models of AGE accumulation and entered into clinical trials in 1996. Despite one phase III clinical trial in which aminoguanidine was shown to reduce the progression of diabetic retinopathy and lower low-density lipoprotein (LDL) and triglyceride levels, a further trial was discontinued due to both a lack of efficacy and safety concerns. Aminoguanidine is reported to result in several side effects including the following: flu-like symptoms, gastrointestinal disturbances, and anemia, and is not currently being investigated for the treatment of AGEs [139]. Besides inhibiting AGE formation, aminoguanidine has also has been shown to be immunosuppressive by inhibiting the enzyme nitric oxide synthase, unrelated to AGE inhibition. Furthermore, the newest experimental anti-AGE drug, ALT-711, failed in human clinical trials due to an inability to reduce AGE levels in vivo.

Our laboratory has shown that first generation bis-2-amnoimidazole (B-2-AI)-derived compounds exhibit potent anti-glycating activity in vitro and are ×100 more active as anti-glycating compounds than aminoguanidine [140]. One lead compound inhibited glycation by 65 % when bovine serum albumin was treated with the potent glycating agent glycolaldehyde. In comparison, aminoguanidine inhibited 35 % AGE formation by glycolaldehyde. We have previously shown that mono 2-aminoimidazoles are non-toxic to mammalian cells and model organisms [141]. These data generated from first generation compounds demonstrate that B-2-AI derivatives have therapeutic potential as potent anti-glycating compounds that may have value as adjunctive treatment for TB. More recently, we have demonstrated that second generation compounds exhibit increased AGE inhibition and breaking activity in a series of in vitro screening assays [142]. Small molecules that inhibit or break preformed AGEs as a by-product of chronic inflammation and altered host metabolism have clinical potential as adjunctive granuloma-targeted therapy.

## The promotion of healing: a balance between extracellular matrix destruction and production

Another approach to therapeutically targeting TB granulomas, besides limiting the destructive inflammatory response, is to promote tissue healing. The processes related to the resolution and repair of damaged tissues occur simultaneously with TB granuloma formation. The rate and extent of granuloma regression is determined by the balance between the mediators of tissue destruction and the factors that oppose or regulate those mediators [143]. Much attention has been given to matrix metalloproteinase (MMP) activity in TB pathogenesis and specifically lesion cavity formation, which is among the most severe manifestations of active TB disease [144]. MMPs are zinc-dependent proteases that degrade a variety of proteins that comprise the extracellular matrix [145]. In humans, there are 23 different MMPs described, several that have been shown to be important in TB pathogenesis. The proteins that have been specifically linked to Mtb infection include MMP-1, MMP-3, MMP-7, MMP-8, MMP-9, MMP-10, and others [146–151]. MMPs are produced by a variety of cell types including inflammatory and immune cells, fibroblasts, and epithelial cells. In addition, several Mtb proteins have been shown to have direct MMP functions or to activate host MMPs, which are secreted as inactive zymogens with a pro-peptide domain, such that extracellular proteolytic cleavage is required for biological activity [145]. MMPs are important in the early stages of protective granuloma formation, but have also been linked to early granuloma as well as TB cavity formation [152]. The extracellular activation of MMPs is in part regulated by tissue inhibitors of metalloproteinases (TIMPs), which are endogenous protease inhibitors that regulate MMP function. Quantitation of circulating and sputum MMPs has been proposed as biomarkers of active TB disease and polymorphisms in MMP genes have been linked to Mtb susceptibility [153, 154]. Moreover, recent studies have suggested that there are gender differences in the expression of MMPs in patients with active TB disease [155].

The role MMPs play in the pathogenesis of a wide variety of communicable and non-communicable diseases such as cardiovascular disease has driven the development of synthetic inhibitors that have potential as adjunctive therapies in the treatment of TB. Since MMPs are zinc-dependent, specific inhibitors have been designed to target or chelate the zinc ion as either broad spectrum or selective inhibitors and several are in various stages of clinical trials. The antimicrobial drug doxycycline at subantimicrobial doses has been shown to inhibit MMPs and currently is the only drug that is clinically available for this purpose. Recent studies have demonstrated that neutrophil-derived MMP-8 was highly expressed in NETs within cavities of TB patients and that doxycycline was effective at limiting collagen destruction by Mtb-mediated MMP-8 [156]. Doxycycline has been shown to not only have antimicrobial activity in vitro but to also reduce the bacterial burden of guinea pigs infected with Mtb [157]. These and other data demonstrate that currently available and approved drugs can serve to limit the destructive consequences of TB granuloma formation as adjunctive therapy. However, because MMPs aid immune effector cell migration and function at the site of infection, the use of this approach is also limited by the ability to time administration to gain the greatest therapeutic potential [158].

Promoting tissue repair and healing can also be accomplished by therapeutic modulation of extracellular matrix lysis as a strategy to limit the unfavorable consequences of Mtb infection. Much is known about the relative expression and immunomodulatory functions of the major cytokines and chemokines associated with Mtb infection in humans and animals. The cytokine network is complex and reflects the simultaneous and dynamic interplay between immune cell stimulation and down regulation and thus the balance between disease progression and resolution [159]. The expression of transforming growth factor β (TGF-β) and other immunosuppressive cytokines has been studied extensively as major immune modulators and as biomarkers for active TB and response to antimicrobial drug treatment [160–165]. Inhibition of TGF-β in particular has been proposed as an attractive therapeutic target to minimize the negative effect on protective immune responses [166, 167]. However, TGF-β is a major fibroblast growth factor and is critical to stimulating the necessary extracellular matrix proteins needed to repair tissue damaged by Mtb infection [168]. TGF-β is produced by numerous different cell types and is considered an anti-inflammatory cytokine with diverse biological functions. Among the most important immunomodulatory functions is the inhibition of immune cell proliferation and regulation of CD4+ T cell differentiation into Foxp3+ regulatory T cells and Th17 cells that are thought to limit the anti-TB effector cell functions. TGF-β gene polymorphisms have been linked to Mtb susceptibility in humans [169, 170]. Moreover, the lack of TGF-β expression or inhibition with drugs or therapeutic antibodies has been shown to be an effective immumunomodulatory strategy in vitro and in vivo [159, 169, 171–173]. The negative consequences of inhibiting TGF-β and other similar growth factors however may be a delay in lesion healing and may inadvertently prolong TB disease and Mtb persistence [174]. Therefore, similar to other therapeutic approaches designed to shift the balance in favor of a more protective immune response, the disadvantages are that other non-immune yet protective host responses will be negatively impacted [175]. Little is known about the impact these strategies will have on the overall resolution of TB disease and more research is needed to adequately weigh the overall benefits, taking into account the full spectrum of host responses to Mtb infection.

## Concluding remarks and future directions

Current opinion on the functional role of the TB granuloma and subsequent approaches to host-directed therapies which target the granuloma are still evolving. Mtb-host interactions within the granuloma remain complex and it is still unclear if the granuloma is protective or destructive to the host. Further, very little is known regarding how a severe necrotic lesion would respond to therapy in comparison to developing lesion at an earlier stage of disease. This review sought to discuss multiple approaches to host-directed therapy in the form of angiogenic, metabolic, AGE, and extracellular matrix modulators. Further research is needed to gain more information about the efficacy of these therapeutic approaches. Also, in vivo animal models will need to be utilized in order to assess the overall effectiveness of the host-directed therapies discussed here as adjunctive treatment with conventional antitubercular agents. The heterogeneity of lesion presentation and range of lesion severity within an individual will likely have an effect on host-directed therapeutic options as well. The need to develop therapies to be applied in conjunction with current antimicrobials remains apparent. This review sought to summarize and explore current as well as novel avenues for host-directed therapies targeting the TB granuloma. Finding effective ways to balance the granulomatous inflammatory response will be on the forefront of the development of host-directed therapies for TB.
